# Formula of compressibility and using it for air, noble gases, some hydrocarbons gases, some diatomic simple gases and some other fluids

**DOI:** 10.1186/s13065-020-00702-5

**Published:** 2020-08-09

**Authors:** Marwan Al-Raeei, Moustafa Sayem El-Daher

**Affiliations:** 1 Faculty of Sciences, Damascus University, Damascus, Syrian Arab Republic; 2 Faculty of Informatics and Communications, Arab International University, Daraa, Syrian Arab Republic; 3 Higher Institute of Laser Applications and Researches, Damascus University, Damascus, Syrian Arab Republic

**Keywords:** Compressibility, Lenard–Jones potential, Bulk modulus, One component fluid, Bulk modulus, Static structure factor, Ornstein–zernike equation and radial distribution function, Speed of sound, Critical temperature, Simple fluid

## Abstract

Based on solutions of the Ornstein–Zernike equation (OZE) of Lennard–Jones potential for mean spherical approximation (MSA), we derive analytical formula for the compressibility assuming that the system is of low density, homogeneous, isotropic and composed of one component. Depending on this formula, we find the values of the bulk modulus and the compressibility of air at room temperature and the bulk modulus and the compressibility of Methane, Ethylene, Propylene and Propane at nine per ten of critical temperature of each hydrocarbon. Also, we find the speed of sound in the air at various temperatures, the speed of sound in each of Helium, Neon, Argon, Krypton, Xenon, Methane, Ethylene, Propylene, Propane, Hydrogen, Nitrogen, Fluorine, Chlorine, Oxygen, Nitrous oxide (laughing gas), Carbon dioxide, Nitric oxide, Carbon monoxide, Sulphur dioxide and dichlorodifluoromethane at room temperature. Besides, we find the speed of sound in Methane, Ethylene, Propylene and Propane at nine per ten of critical temperature of each hydrocarbons depending on the formula we find. We show that the simple formula we derive in this work is reliable and agrees with the results obtained from other studies and literatures. We believe it can be used for many systems which are in low densities and described by Lennard–Jones potential.

## Background

The compressibility is one of the most important properties in thermodynamic of materials, and we can get it from experimental methods or from some theoretical methods. In this work we find analytical formula of the compressibility from the Ornstein–Zernike equation which is one of the basic equations used to study the physical properties of fluids because this equation enables us to find the physical properties of materials by theoretical ways. For one component system, the Ornstein–Zernike equation in the homogeneous formalism is given as follows [1–7]:

1 $$ h(r) = c(r) + \rho \int {d\vec{r}^{\prime}c(\left| {\vec{r} - \vec{r}^{\prime}} \right|)} h(r^{\prime}) $$

where *c*(*r*) is the direct correlation function, *h*(*r*) is the total correlation function, *ρ* is particle’s density and *r* is the position and the integral is over the volume of position of the particles. The Ornstein–Zernike equation is considered a very important equation in the statistical mechanics and materials sciences, especially, in the static formalism because by solving this equation we find the radial distribution function (RDF) of a specific system which enables us to find a lot of properties of the material by applying the integration of a certain property on this function. We can find a solution for the Ornstein–Zernike equation using a suitable interaction potential of the system, however, we need another equation between pair potential and the total correlation function or the direct correlation function which we get it from a number of possible approximations of the direct correlation function which are used in the theory of simple liquids or simple fluids such as Born Green Yvon approximation (BGYA), Hyper Netted Chain approximation (HNCA), Percus Yevick approximation (PYA) and mean spherical approximation (MSA). All of these approximations give closed relations between the direct correlation function and the interaction potential of the system either in a linear form or in a nonlinear form [8–25]. In this work, we use the mean spherical approximation to find the solutions of the Ornstein–Zernike equation where this approximation relates the direct correlation function and the interaction potential via a linear formula. The direct correlation function based on the mean spherical approximation is given as follows [2, 4–7]:

2 $$ c(r) \approx - U(r)/(k_{B} T)\begin{array}{*{20}c} {} & ; & {} & {r > d} \\ \end{array} $$

where $k_{B}$ is Boltzmann constant, *T* is absolute temperature and *d* is the diameter of particles while *U*(*r*) is the interaction potential between the particles of the system. The interaction potential which we used in this work is Lenard–Jones potential, which is very important as a fitting potential and a structure potential in a lot of studies such as soft materials and simple fluids [3, 8–24] and this potential is given by the following formula:

3 $$ U_{LJ} (r) = 4\varepsilon \left[ {(\frac{\sigma }{r})^{12} - (\frac{\sigma }{r})^{6} } \right] = \varepsilon \left[ {(\frac{{r_{m} }}{r})^{12} - 2(\frac{{r_{m} }}{r})^{6} } \right] $$

where *ε* represents the depth of Lenard–Jones potential or its minimum value and *r*_*m*_ is the distance at which Lenard–Jones potential equals its minimum value which is called the minimum distance of Lenard–Jones potential while *σ* is the distance at which Lenard–Jones potential equals zero.

## Methods

We find a formula for the compressibility of one component fluid from the solutions of the Ornstein–Zernike equation for Lenard–Jones potential using mean spherical approximation for the direct correlation function. We obtain the radial distribution function of the system and from this function we get the compressibility of the system which is related to the radial distribution function via the following formula [1, 7, 11]:

4 $$ \frac{{\chi_{T} }}{{\chi_{T}^{id} }} = 1 + \rho \int {g(r)d\vec{r} - } \rho \int {d\vec{r}} $$

where *β* = *1*/(*k*_*B*_*T*), $\chi_{T}^{id}$ is the compressibility of ideal gas and *g(r)* is the radial distribution function of the system. So, If we use the solutions of the Ornstein–Zernike equation of Lenard-Johns potential from mean spherical approximation in the previous equation and if we use the integral of the position instead of the integral of the volume in the homogeneous and isotropic case, we find that the compressibility of the system is given by the following integral equation:

5 $$ \chi_{T} = \chi_{T}^{id} - C_{1} \int\limits_{0}^{d} {r^{2} dr - C_{2} \int\limits_{d}^{\infty } {[\alpha^{2} \frac{d}{{r^{10} }}^{12} - \alpha \frac{d}{{r^{4} }}^{6} ]dr} } $$

where *C*_1_, *C*_2_ are coefficients and *α* is defined as follows:

6 $$ \alpha = (1 + \sqrt {1 + 1/\beta \varepsilon } )/2 $$

By integrating the equation of the compressibility over the position, we find the following formula of the compressibility:

7 $$ \chi_{T} = \left[ {1 - \frac{4}{3}\pi \rho \left( {1 - \alpha \beta U_{0} + \frac{{\alpha^{2} }}{3}\beta U_{0} } \right)d^{3} } \right]\chi_{T}^{id} ;\quad U_{0} = 4\varepsilon $$

## Results and discussion

The previous equation represents the basic thing of this study which is the formula of the compressibility. We see that the formula of the compressibility that we found (Eq. 7) contains the Lennard–Jones potential parameters, the diameter of particles in the system, the temperature and the density of the system’s particles. We can use the formula in a wide variety of materials interacting with each other via Lennard–Jones potential such as light polymers and some simple fluids systems such as atomic Argon. In this work, we use this formula to calculate the compressibility and the bulk modulus for some hydrocarbons and air. Besides and based on the formula, we calculate the speed of sound in some atomic fluids such as Argon, some hydrocarbons, diatomic fluid such as Oxygen and some other gases such as dichlorodifluoromethane. We calculated the compressibility and the bulk modulus of air from this study, i.e. Eq. 7, at 298.16 K° and we inserted the results in Table 1 with the value of bulk modulus of air found in some literatures in addition to the Lenard–Jones potential’s parameters of air.

**Table 1:** The compressibility and the bulk modulus of air Β from Eq. 7 and the bulk modulus of air from the literatures Β* at 25 °C

$$\begin{gathered} \sigma \\ ({\rm A}^{ \circ } ) \\ \end{gathered}$$	$$\begin{array}{*{20}c} \varepsilon & { \times 10^{2} } \\ \end{array}$$$$(ev)$$	$$T$$$$(K^{ \circ } )$$	$$\begin{gathered} \chi_{\rm T} \\ ({\text{MPa}}^{ - 1} ) \\ \end{gathered}$$	$$\begin{gathered} {\rm B} \\ ({\text{MPa}}) \\ \end{gathered}$$	$$\begin{gathered} {\rm B}^{*} \\ ({\text{MPa}}) \\ \end{gathered}$$
3.6170	1.033	298.16	9.7929	0.1021	0.1010

As we see from Table 1, the result resulted from this work and the result found in the literatures for the bulk modulus of air are close to each other at the previous temperature.

In addition to that, we calculated the compressibility of air from the formula we derived in this work at different temperatures and we inserted the results of this calculation in Table 2. With the bulk modulus of air at the same temperatures. As we see from Table 2, the bulk modulus of air increases when temperature increases which agree well with literatures.

**Table 2:** The compressibility of air and the bulk modulus of air based on Eq. 7 at different temperatures in the gaseous phase

$$\begin{gathered} t \\ (C^{ \circ } ) \\ \end{gathered}$$	$$\begin{gathered} \rho_{m} \\ (mg/cc) \\ \end{gathered}$$	$$\begin{gathered} \chi_{\rm T} \\ ({\text{MPa}}^{ - 1} ) \\ \end{gathered}$$	$$\begin{gathered} {\rm B} \times 10 \\ ({\text{MPa}}) \\ \end{gathered}$$	$$\begin{gathered} t \\ (C^{ \circ } ) \\ \end{gathered}$$	$$\begin{gathered} \rho_{m} \\ (mg/cc) \\ \end{gathered}$$	$$\begin{gathered} \chi_{\rm T} \\ ({\text{MPa}}^{ - 1} ) \\ \end{gathered}$$	$$\begin{gathered} {\rm B} \times 10 \\ ({\text{MPa}}) \\ \end{gathered}$$
−25	1.4224	9.8726	1.0129	5	1.2844	9.7480	1.0259
−24	1.4178	9.8647	1.0137	6	1.2798	9.7478	1.0259
−23	1.4132	9.8570	1.0145	7	1.2752	9.7479	1.0259
−22	1.4086	9.8495	1.0153	8	1.2706	9.7483	1.0258
−21	1.4040	9.8424	1.0160	9	1.2660	9.7488	1.0258
−20	1.3994	9.8355	1.0167	10	1.2614	9.7497	1.0257
−19	1.3948	9.8289	1.0174	11	1.2568	9.7508	1.0256
−18	1.3902	9.8225	1.0181	12	1.2522	9.7521	1.0254
−17	1.3856	9.8164	1.0187	13	1.2476	9.7537	1.0252
−16	1.3810	9.8106	1.0193	14	1.2430	9.7556	1.0251
−15	1.3764	9.8050	1.0199	15	1.2384	9.7577	1.0248
−14	1.3718	9.7997	1.0204	16	1.2338	9.7601	1.0246
−13	1.3672	9.7947	1.0210	17	1.2292	9.7627	1.0243
−12	1.3626	9.7899	1.0215	18	1.2246	9.7656	1.0240
−11	1.3580	9.7854	1.0219	19	1.2200	9.7687	1.0237
−10	1.3534	9.7811	1.0224	20	1.2154	9.7721	1.0233
−9	1.3488	9.7771	1.0228	21	1.2108	9.7757	1.0229
−8	1.3442	9.7734	1.0232	22	1.2062	9.7796	1.0225
−7	1.3396	9.7699	1.0236	23	1.2016	9.7838	1.0221
−6	1.3350	9.7667	1.0239	24	1.1970	9.7882	1.0216
−5	1.3304	9.7637	1.0242	25	1.1924	9.7929	1.0211
−4	1.3258	9.7610	1.0245	26	1.1878	9.7978	1.0206
−3	1.3212	9.7585	1.0247	27	1.1832	9.8030	1.0201
−2	1.3166	9.7563	1.0250	28	1.1786	9.8085	1.0195
−1	1.3120	9.7544	1.0252	29	1.1740	9.8142	1.0189
0	1.3074	9.7527	1.0254	30	1.1694	9.8202	1.0183
1	1.3028	9.7512	1.0255	31	1.1648	9.8265	1.0177
2	1.2982	9.7501	1.0256	32	1.1602	9.8330	1.0170
3	1.2936	9.7491	1.0257	33	1.1556	9.8398	1.0163
4	1.2890	9.7484	1.0258	34	1.1510	9.8468	1.0156

Also, We calculated the speeds of sound in some inert gases (Helium, Neon, Argon, Krypton and Xenon) based on the formula which we found and the results were illustrated in Table 3 with the densities, the molar masses and Lenard–Jones potential parameters of the noble gases.

**Table 3:** The speeds of sound in noble gases at t = 25 °C from this work based on Eq. 7

Substance	$He$	$Ne$	$Ar$	$Kr$	$Xe$
$\rho_{m} (mg/cc)$	0.1786	0.9002	1.7840	3.7490	5.8940
$\sigma ({\rm A}^{ \circ } )$	2.576	2.789	3.432	3.675	4.009
$\varepsilon /k_{B} (K^{ \circ } )$	10.2	35.7	122.4	170.0	234.7
$M(g/mol)$	4.0026	20.1797	39.7920	83.7980	131.2930
$v(m/s)$	787.4806	350.7260	249.5060	171.7220	136.8410

As we see from Table 3, the values of the speed of sound of the noble atomic gases which we calculated from this study based on the simple formula that we found have the same order with other references [26–30] for the gaseous Helium, references [26, 30] for the gaseous Neon, references [26–28, 30] for the gaseous Argon and references [28, 30] for the gaseous Krypton and the gaseous Xenon. We see that the smallest value of the speed of sound is for Xenon and the biggest value is for Helium which also agrees with literatures.

Also, We calculated the speeds of sound in some hydrocarbons (Methane, Ethylene, Propylene and Propane) from this work, based on Eq. 7, because these hydrocarbons interact through Lenard–Jones potential like in [31], the results were inserted in Table 4 with the densities, the molar masses and Lenard–Jones potential’s parameters of the used hydrocarbon materials. We used the previous hydrocarbons in the calculations of the compressibility and the bulk modulus as an example of other hydrocarbons and because the parameters of the interaction potential are known for these hydrocarbons and we can compare the bulk modules values of these hydrocarbons with other studies.

**Table 4:** The speeds of sound in Methane, Ethylene, Propylene and Propane at t = 25 °C from this work based on Eq. 7

Hydrocarbon	$CH_{4}$	$C_{2} H_{4}$	$C_{3} H_{6}$	$C_{3} H_{8}$
$\rho_{m} (mg/cc)$	0.657	1.18	1.81	2.01
$\sigma ({\rm A}^{ \circ } )$	3.780	4.228	4.766	4.934
$\varepsilon /k_{B} (K^{ \circ } )$	1.31	1.84	2.34	2.33
$M(g/mol)$	16.04	28.05	42.08	44.10
$v(m/s)$	392.6560	296.1230	240.6070	234.6880

We see from Table 4 that the speed of sound agrees well with other references, references [26–28, 30] for the gaseous Methane and the gaseous Ethylene, reference [30] for the gaseous Propylene, references [28, 30] for the gaseous Propane at 25 °C. In addition, we calculated the compressibility of the same hydrocarbons at temperatures equal to 0.9 of the critical temperature *T*_*C*_ and pressures about 0.5 of critical pressure *P*_*C*_ of each hydrocarbon from this study**, **i.e. Equation 7, and we inserted the results in the Table 5 which also, contains Lenard–Jones potential’s parameters of these hydrocarbon materials in addition to the molar mass of the hydrocarbons. For comparison our results with other results, we calculated the bulk modulus at the previous temperatures for these hydrocarbons and we inserted the results with the results for the bulk modulus of these hydrocarbons at the previous conditions from reference [32] in Table 6 which also contains the compressibility from our calculations.

**Table 5:** The compressibility of some hydrocarbons from Eq. 7 at 0.9 *TC* of each hydrocarbon

Hydrocarbon	$$\begin{gathered} \sigma \\ ({\rm A}^{ \circ } ) \\ \end{gathered}$$	$$\begin{gathered} \varepsilon \times 10^{2} \\ (eV) \\ \end{gathered}$$	$$\begin{gathered} M \\ (g/mol) \\ \end{gathered}$$	$$\begin{gathered} \chi_{T} \\ (atm^{ - 1} ) \\ \end{gathered}$$
$CH_{4}$	3.780	1.31	16.04	0.0425
$C_{2} H_{4}$	4.228	1.84	28.05	0.0450
$C_{3} H_{6}$	4.766	2.34	42.08	0.0794
$C_{3} H_{8}$	4.934	2.33	44.10	0.0340

**Table 6:** The bulk modulus of the some hydrocarbons from our work and from reference [32] at 0.9 *TC* of each hydrocarbon

Hydrocarbon	$$\begin{gathered} \chi_{T} \\ (atm^{ - 1} ) \\ \end{gathered}$$	$$\begin{gathered} {\rm B}^{ThisWork} \\ (atm) \\ \end{gathered}$$	$$\begin{gathered} {\rm B}^{[32]} \\ (atm) \\ \end{gathered}[32]$$
$CH_{4}$	0.0425	23.5294	29.615
$C_{2} H_{4}$	0.0450	22.2222	–
$C_{3} H_{6}$	0.0794	12.5945	–
$C_{3} H_{8}$	0.0340	29.4118	39.487

We calculated the speeds of sound in the same hydrocarbons at the same conditions from this study and the results were inserted in Table 7 with comparisons from reference [30] for the speeds of sound in the same hydrocarbons.

**Table 7:** The speeds of sound in the last hydrocarbons from our work and from reference [30] at the same previous conditions

Hydrocarbon	$$\begin{gathered} \chi_{T} \\ (atm^{ - 1} ) \\ \end{gathered}$$	$$\begin{gathered} v^{ThisWork} \\ (m/s) \\ \end{gathered}$$	$$\begin{gathered} v^{[30]} \\ (m/s) \\ \end{gathered}$$
$CH_{4}$	0.0425	244.2184	277.62
$C_{2} H_{4}$	0.0450	234.7020	257.79
$C_{3} H_{6}$	0.0794	232.9465	239.00
$C_{3} H_{8}$	0.0340	192.8914	194.37

As we note from the comparisons between the values of the bulk modulus of Methane and the bulk modulus of Propane which we calculated from this study with the values of the bulk modulus of Methane and the bulk modulus of Propane resulted from reference [32] at the same conditions in Table 6, the values are of the same order and close to each other.

Also, we see the same thing from the comparisons between the values of the speed of sound in the four hydrocarbons calculated from this study and within reference [30] in Table 7 at the same conditions. After that, we calculated the values of the speed of sound in some simple diatoms gases, namely, Hydrogen, Nitrogen, Fluorine, Chlorine and Oxygen from this study, i.e. Eq. 7, and we inserted the results in Table 8. The densities, the molar masses and Lenard–Jones potential parameters of the considered diatomic simple gaseous materials were inserted in the same table.

**Table 8:** The speeds of sound in Hydrogen, Nitrogen, Fluorine, Chlorine and Oxygen at t = 25 °C from this study based on Eq. 7 and from references [26–28, 30]

Substance	$H_{2}$	$N_{2}$	$F_{2}$	$Cl_{2}$	$O_{2}$
$\rho_{m} (mg/cc)$	0.0823	1.1452	1.5537	3.2000	1.3087
$\sigma ({\rm A}^{ \circ } )$	2.915	3.667	3.653	4.115	3.433
$\varepsilon /k_{B} (K^{ \circ } )$	38.0	99.8	112.0	357.0	113.0
$M(g/mol)$	2.0159	28.0134	37.9968	70.9060	31.9988
$v(m/s)$	1109.7000	297.4974	255.3772	185.3550	278.2920

As we see from Table 8, the values of speed of sound in the previous diatomic simple gases which calculated from this study and the values in other studies, references [26–28, 30] for the gaseous Hydrogen and the gaseous Oxygen, references [26, 27, 30] for the gaseous Nitrogen, reference [30] for the gaseous Fluorine and references [26, 27] for the gaseous Chlorine, have the same order. Besides, we see that the biggest value of the speed of sound is for the Hydrogen and the smallest value is for the Chlorine. Finally, we calculated the values of the speed of sound in some gaseous oxides (Nitrous oxide, Carbon dioxide, Nitric oxide, Carbon monoxide and Sulphur dioxide) in addition to the speed of sound in dichlorodifluoromethane. We inserted the results for the previous gases in Table 9. The densities, the molar masses and Lenard–Jones potential parameters of these gaseous materials were inserted in the same table.

**Table 9:** The speeds of sound in Nitrous oxide, Carbon dioxide, Nitric oxide, Carbon monoxide, Sulphur dioxide and Dichlorodifluoromethane at t = 25 °C from this work based on Eq. 7

Substance	$N_{2} O$	$CO_{2}$	$NO$	$CO$	$SO_{2}$	$CCl_{2} F_{2}$
$\rho_{m} (mg/cc)$	1.8088	1.8079	1.3402	1.1453	2.6642	2.0383
$\sigma ({\rm A}^{ \circ } )$	3.879	3.996	3.470	3.590	4.026	5.116
$\varepsilon /k_{B} (K^{ \circ } )$	220.0	190.0	119.0	110.0	363.0	280.0
$M(g/mol)$	44.0128	44.0095	30.0061	28.0101	64.0640	120.9140
$v(m/s)$	236.6072	236.7511	287.3415	297.4551	195.2006	142.5648

We see that the values of the speed of sound in the previous gases (Table 9) agree with the results from references [26, 28] for the gaseous Nitrous oxide and the gaseous Carbon monoxide, references [26, 28, 30] for the gaseous Carbon dioxide and the gaseous Sulphur dioxide, reference [26] for the gaseous Nitric oxide and reference [30] for the gaseous dichlorodifluoromethane.

## Conclusion

In this work, we derived analytical formula for the compressibility for homogenous and isotropic system composed of one component at low density assuming that the particles in the system interact each other via Lenard-Jones potential which contains two parts, the first part is repulsive and the other is attractive. The compressibility can be found from some experimental methods such as [33] and some theoretical methods such as virial expansion [34, 35]. In this work, we found a formula of the compressibility as a function of particle’s density, Lenard–Jones potential parameters and the temperature based on solutions of the Ornstein–Zernike equation for mean spherical approximation.

The formula we derived was employed to find the compressibility and the bulk modulus values of air at 25 °C (Tables 1 and 2) and of some hydrocarbons at defined temperatures of each hydrocarbon (Tables 5 and 6), the results of the bulk modulus and the compressibility found from this study agree qualitatively with the literature for air and other reference [32] for hydrocarbons. Besides, the speeds of sound in some hydrocarbons at defined temperatures of each hydrocarbon (Tables 4 and 7) and the speeds of sound in Helium, Neon, Argon, Krypton, Xenon, Hydrogen, Nitrogen, Oxygen, Chlorine, Fluorine, Methane, Ethylene, Propylene, Propane, Carbon monoxide, Carbon dioxide, Sulfur dioxide, Laughing gas, Nitric oxide and dichlorodifluoromethane (Tables 3, 8 and 9). We found that our results agree qualitatively with other studies.

The formula that we derived for the compressibility (Eq. 7) is simple and it can be applied for many fluids that interact via Lenard–Jones potential, only, we need the Lenard-Jones potential parameters and the density of particles in the system at a certain temperature.

## Data Availability

We declared that the materials in the paper will be available for non-commercial purposes and the corresponding author, M Al-Raeei, must be contacted for requesting the data.

## Abbreviations

OZE: Ornstein–Zernike Equation
MSA: Mean Spherical Approximation
BGYA: Born Green Yvon Approximation
HNCA: Hyper Netted Chain Approximation
RDF: Radial Distribution Function
PYA: Percus Yevick Approximation
